# Successful soft and hard tissue augmentation with platelet‐rich fibrin in combination with bovine bone space maintainer in a delayed implant placement protocol in the esthetic zone: A case report

**DOI:** 10.1002/ccr3.2177

**Published:** 2019-05-08

**Authors:** Joost E. I. G. Brouwers, Sharon Buis, Rianne Haumann, Philip Ph. G. de Groot, Bas de Laat, Jasper A. Remijn

**Affiliations:** ^1^ Institute for Dental Implantology Amersfoort The Netherlands; ^2^ Department of Clinical Chemistry and Hematology Gelre Hospitals Apeldoorn The Netherlands; ^3^ Synapse Research Institute Maastricht The Netherlands

**Keywords:** bone augmentation, growth factor release, platelet‐rich fibrin, soft tissue augmentation, wound healing

## Abstract

Replacement in the esthetic zone can be very unpredictable and difficult to manage in cases with extreme bone and soft tissue loss. In this case report (2.5‐year follow‐up), we demonstrate that the use of platelet‐rich fibrin in combination with bovine bone can result in a stable, esthetic outcome.

## 1 INTRODUCTION

The anterior maxilla region is an anatomically difficult region for dental implantation. Soft and hard tissue augmentations are often needed to restore the affected site. A sufficient bone density and volume is needed for stable placement of dental implants.^1^, ^2^, ^3^ In addition, esthetic outcome is an important parameter for the patient. The main esthetic objective for patients is to maintain a harmonious gingival contour with intact papillae and without abrupt changes.^1^, ^4^

Placement of dental implants in the anterior maxillary region can be achieved by different methods.^4^ The optimal method is dependent on anatomical parameters such as bone volume, bone density, alveolar crest position, adjacent teeth, and gingival morphology. Moreover, esthetic outcomes are important for successful dental implantation which are determined by the smile and lip line.^1^, ^3^

In order to increase the chance of successful dental implantation sufficient bone volume and quality are needed. Bone augmentation can be achieved by several different methods such as autologous bone grafting, xenogenous and alloplastic bone grafting, guided bone regeneration (GBR), and distraction osteogenesis.^1^, ^3^ Insufficient bone, including bone height, thickness, volume, and quality, increases the risk of implant failure due to inadequate implant stability.^1^, ^3^ The bone is also a scaffold for soft tissue; when the implant is placed into bone with inadequate bone height and thickness, harmonious gingival contour is difficult to achieve.^1^ The type of dental implant is especially a challenge in the anterior maxillary region. The choice of the dental implant is dependent on the bone volume and quality. An implant is often successfully placed in bone that has a width of 5 mm and a height of 7 mm.^5^ Implants that are frequently used in the anterior maxilla are tapered implants, because of their ability to achieve a higher initial stability in spongeous bone.^6^

Gingiva is often affected, and soft tissue augmentation is needed to restore the harmonious gingiva line.^1^ The type of gingiva influences the treatment method used for the repair of gingival recession.^7^ Common approaches are free gingival grafts, coronally positioned flap, subepithelial connective tissue grafts, acellular dermal grafts, and enamel matrix proteins.^7^ The subepithelial connective tissue grafts are generally considered as the “gold standard” in gingival augmentation.^7^

Bone height and thickness are important for long‐term stability of harmonious gingival margins around implants and adjacent teeth.^1^ Loss of buccal bone around the implant frequently results in soft tissue recession potentially exposing implant collars and leading to loss of the harmonious gingival margin and horizontal or vertical bone loss of the alveolar crest.^1^ The gingival morphology is important and can be divided into a thin highly scalloped gingiva or thick with shallow scalloped gingiva.^1^

We have used platelet‐rich fibrin (PRF) successfully in our clinic for different applications such as bone and soft tissue augmentation, periodontal pocket reduction surgery, soft tissue dehiscence coverage, and in combination with surgical removal of wisdom teeth. Autologous PRF is prepared from the patient's blood using a dedicated centrifugation protocol.^8^ PRF consists of a polymerized fibrin network containing platelets and sometimes white blood cells (depending on the used protocol).^8^, ^9^ The membrane releases growth factors that influence the wound healing process.^10^, ^11^, ^12^, ^13^, ^14^ PRF can be applied in both hard and soft tissue augmentations. The benefit of PRF compared with standard procedures is the reduction in bone augmentation time.^15^ For soft tissue augmentation and remodeling of the gingiva, the PRF membrane is especially placed in which strong fibrin architecture could be used as a matrix for wound repair.^10^ In this case report, we describe application of PRF for multiple procedures in a challenging case of hard and soft tissue deficiency in the anterior maxilla region.

## 2 THE CASE

**Figure 1:**
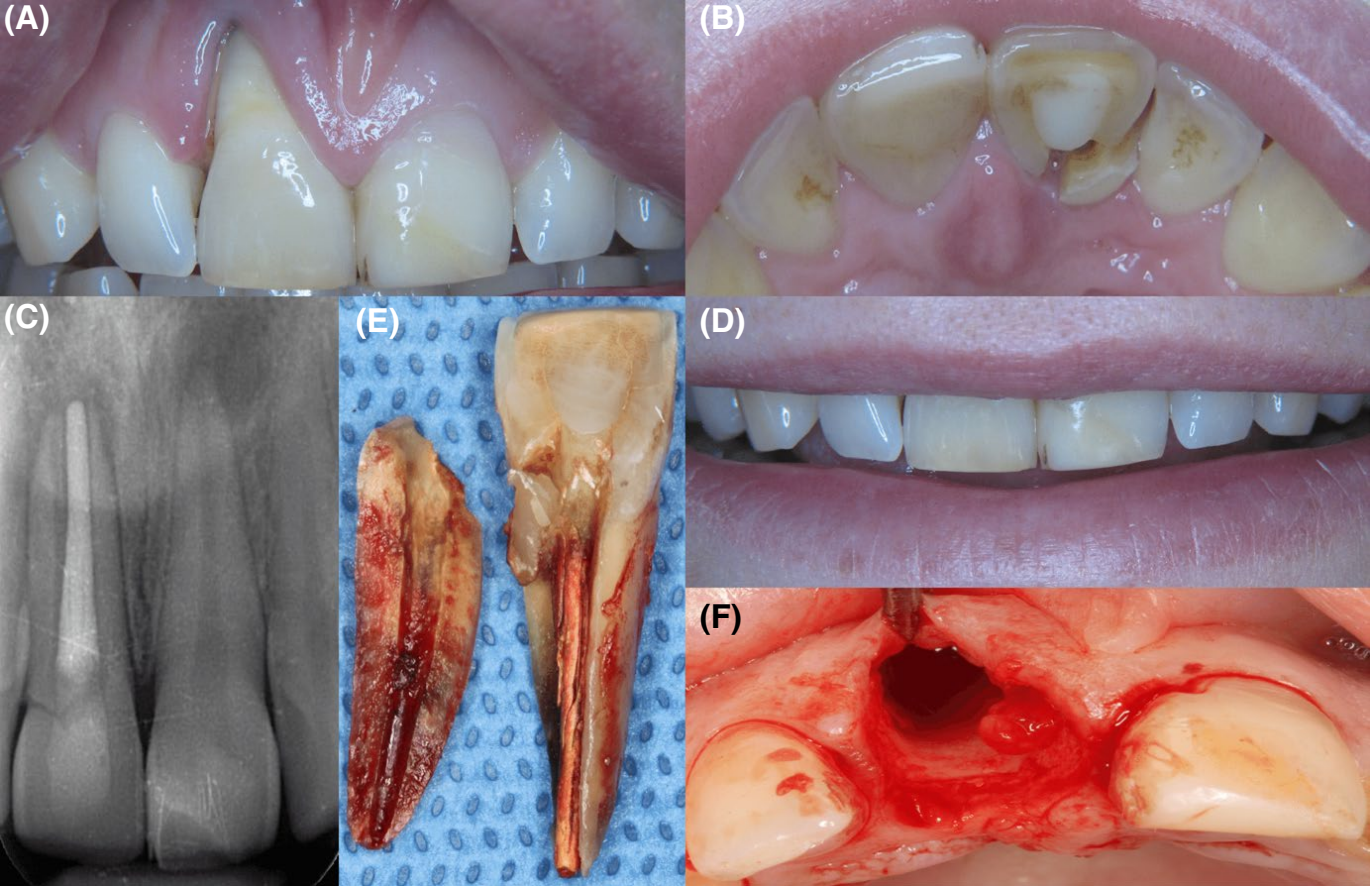
Esthetic and radiographic overview of the failing right upper central incisor. A and B, clearly shows the hard and soft tissue deficiency and the fracture line, respectively. C, shows the radiologic assessment of failing tooth number 11 and fracture line. The patient's smile line was not visibly affected by the tissue deficiency. D, shows the patient's smile line. E, shows the extracted tooth, and F, shows both the hard and soft tissue deficiencies after tooth extraction

A healthy woman (ASA‐score I), 27 years of age, presented with a failing right upper central incisor (tooth number 11). The incisor was fractured in a vertical direction due to a field hockey injury 10 years ago. The patient had an endodontic treatment at the time of the injury on tooth number 11. Today, an enormous gingival deficiency on the buccal site was present and at the palatal side the fracture was clearly present (Figure 1A,B). Radiographic inspection confirmed the endodontic treatment and demonstrated the fracture line (Figure 1C). Furthermore, the buccal bone was completely destroyed. The patient had a normal smile line, a thick gingival biotype, and probing depths of the adjacent teeth were within normal limits (Figure 1D).

The tooth could not be salvaged; therefore, the treatment was to replace the central incisor with an implant. The tooth was extracted (Figure 1E), and the severe loss of buccal bone was clearly visible after extraction (Figure 1F). The alveolus was carefully curetted to eliminate any residual infective tissue to prevent compromised osseointegration.

**Figure 2:**
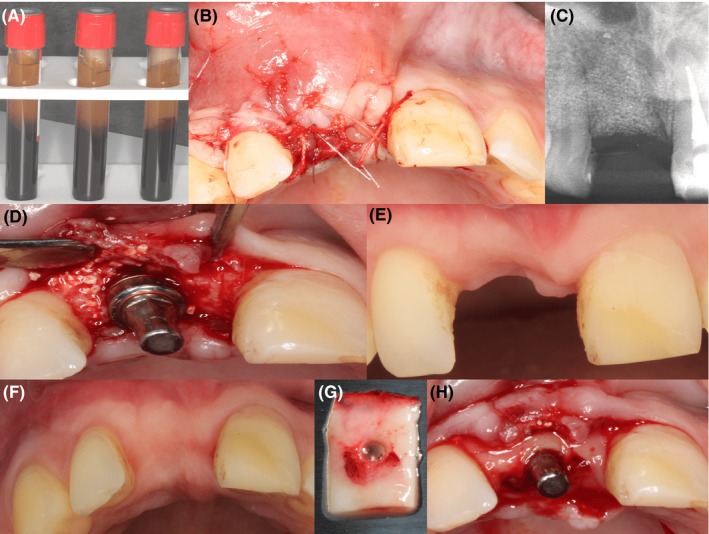
Preparation and the use of the PRF membrane. A, shows the PRF clot obtained after centrifugation. B, shows PRF membrane inserted into the cavity. C, shows the radiologic assessment of deficiency at 3 months after augmentation. D, shows implant placement with immediate temporary abutment. E and F, shows buccal deficiency after the buildup and occlusal appearance after buildup, respectively. G, shows the punched PRF membrane. H, shows PRF membrane placed in the buccal/palatal envelope

Due to the severe bone loss, bone augmentation was needed to reconstruct the buccal bone and increase bone volume to improve chances for successful implantation. Bone augmentation is usually achieved by intraoral autogenous bone augmentation. However, PRF has shown promise above the conventional methods of bone augmentation.^16^, ^17^ The PRF method is thoroughly described^8^; briefly, whole blood is taken in a PRF tube and immediately centrifuged (Process for PRF, Nice, France) at 200 *g* for 8 min.^18^ The PRF tube is left in an upright position for 10 min at room temperature (Figure 2A). The PRF is separated from the red blood clot and pressed in the PRF box for 1 min. The liquid fraction, which was collected after compression, is mixed with the bovine bone space maintainer (BEGO OSS, BEGO Implant Systems, Bremen, Germany). The space maintainer is put into the cavity combined with the PRF liquid fraction and covered with the PRF membrane (Figure 2B). The patient was closely monitored for the next 18 days. The patient showed little swelling, but had no pain. The 3‐month follow‐up showed stable bone augmentation which was radiographically examined by X‐ray (Figure 2C). Endodontic treatment was recommended and executed by the referral dentist on central incisor 21 because of an apical translucency on the central incisor 21 and no vital reaction of the pulp.

After four months, bone augmentation was sufficient for a BEGO Semados^®^ RS implant (BEGO OSS, BEGO Implant Systems), length of 15 and 4.1 mm diameter, placement (Figure 2D). After placement, a primary stability of 30 Ncm was reached which was within the advised range for immediate loading.

**Figure 3:**
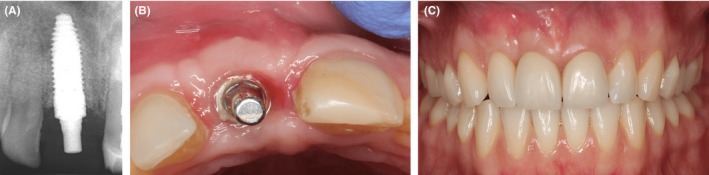
Implant placement. A, shows the radiographic assessment of implant placement 4 weeks after implant insertion. B, shows thickening of the soft tissue 4 weeks after membrane placement. C, shows final restorations in place. Notice the position of the attached gingiva and the zenith

**Figure 4:**
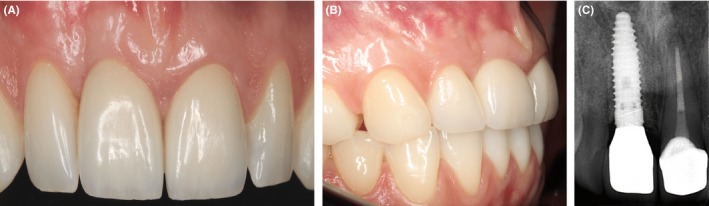
Postoperative assessment of the implant placement after one year. A and B, one‐year post‐op. C, shows the radiographic assessment one‐year post‐op. No change in position of attached gingiva and zenith

**Figure 5:**
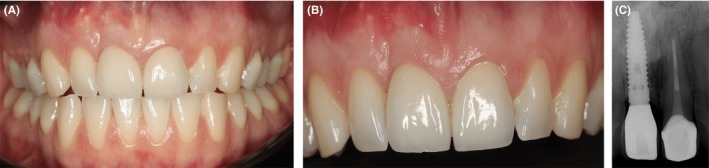
Postoperative assessment of the implant placement after two and a half years. A and B, shows two and a half years post‐op. C, shows the radiographic assessment two and a half years post‐op; note there is no change in soft and hard tissue position

A BEGO Immediate Temporary Abutment (PS ITA) (BEGO OSS, BEGO Implant Systems) was placed on the implant and was restored with a provisional crown. However, there was still insufficient tissue in the anterior maxillary region. From the occlusal perspective, there was a deficiency in the hard and soft tissue contour (Figure 2F but the frontal aspect of the soft tissue was in normal range (Figure 2E). The gingival zenith was initially very apical and became almost symmetrically with the neighboring teeth. PRF was used for thickening of the soft tissue (Figure 2G,H). Four weeks after implant placement, the proper dimensions in buccal and palatal direction were obtained (Figure 3B). Fourteen months after the augmentation of soft and hard tissue, the final crowns were placed on the implant and the central incisor 21 (Figure 3C). Follow‐up after one (Figure 4A,B,C) and two years demonstrates a very stable hard and soft tissue volume (Figure 5A,B,C).

## 3 DISCUSSION

Soft tissue augmentation is especially important in the esthetic zone. The facial soft tissue parts will resorb quite quickly which should be prevented. The interdental papillae will disappear fast after extraction, and in addition, the enormous bone loss will influence the gingiva.^17^ The initial quality of the gingival tissue is important.^19^, ^20^, ^21^, ^22^, ^23^ A thin biotype is less predictable then a thick biotype. The wound should be closed primarily without any tension. If the gingival tissue is weak or damaged, sloughing of the soft tissue is likely to occur and will lead to a compromised healing site due to contamination.

Long‐term clinical studies have shown that functional osseointegration is a predictable outcome. However, the success of dental implant therapy is no longer based only on functional osseointegration but also on positive patient outcomes as esthetic harmony with the remaining dentition. In the present case report, we describe the use of PRF in combination with bone substitute for hard and soft tissue augmentation. We have applied this approach in our clinic for several years, because it may have a stimulating effect on the healing and maturation of soft and osseous tissues.

After extraction of the central incisor, the extend of bone loss was clearly visible. Therefore, immediate loading of the implant was not possible due to the resorption of the alveolar ridge. One of the prerequisites for immediate loading is primary stability, which only can be achieved if there is enough surrounding bone.^24^, ^25^ Without immediate grafting after extraction, the alveolar ridge would resorb immediately, resulting in inadequate bone volume.^26^ In order to increase the bone volume, PRF and bovine bone substitute were used. In previous studies, it was shown that PRF is a suitable technique for bone augmentation.^9^ One of the benefits of PRF is that PRF increases the bone‐to‐implant contact compared with other bone augmentation techniques.^9^ However, some bone augmentation techniques have the potential of angiogenesis; therefore, it cannot be excluded that PRF solely is a superior technique for bone augmentation.^27^ The role of PRF in wound healing has been demonstrated by Agrawal et al^28^ by prolonged release of platelet‐derived growth factors at the wound site, proliferation of fibroblasts and osteoblasts, promoted angiogenesis, induced collagen synthesis, guided wound coverage, mechanical adhesion by fibrin, trapped circulating stem cells, and regulation of immunity. In addition, the membrane acts as a bio‐barrier and an engineering scaffold.^15^

## 4 CONCLUSION

In this case report, we have showed that PRF in combination with bovine bone space maintainer is a promising method for buccal bone augmentation as well as soft tissue restoration. This approach resulted in natural healing and maturation of the peri‐implant bone and soft tissues. Bone augmentation was achieved with enough bone and bone density in order to achieve sufficient implant stability. The patient had a good functional and esthetic outcome. In this case, the application of PRF was a simple, affordable, and accessible method. The approach of PRF in combination with bovine bone substitute may be a promising development in oral implantology, although more knowledge about the molecular properties of PRF is needed for optimal implementation.

## CONFLICT OF INTEREST

The authors declare no conflict of interest.

## AUTHOR CONTRIBUTION

JEIGB: involved in concept/design, data analysis/interpretation, drafting article, data collection, and approval of article. SB: involved in concept/design, data collection, and approval of article. RH: involved in analysis, drafting article, and approval of article. PGdG: involved in concept, critical revision of article, and approval of article. BdL: involved in concept, critical revision of article, and approval of article. JAR: involved in concept/design, analysis, data collection, critical revision of article, and approval of article.

## ETHICAL APPROVAL

A written informed consent was obtained according to the ethical guidelines of the 1975 Declaration of Helsinki (2013).
